# Type 1 and type 2 diabetes after gestational diabetes: a 23 year cohort study

**DOI:** 10.1007/s00125-020-05215-3

**Published:** 2020-07-29

**Authors:** Anna-Maaria Auvinen, Kaisu Luiro, Jari Jokelainen, Ilkka Järvelä, Mikael Knip, Juha Auvinen, Juha S. Tapanainen

**Affiliations:** 1 Department of Obstetrics and Gynecology, Oulu University Hospital, Oulu, Finland; 2 PEDEGO Research Unit, Medical Research Centre, University of Oulu, Oulu, Finland; 3 Department of Obstetrics and Gynecology, University of Helsinki and Helsinki University Hospital, PO 140 (Haartmaninkatu 2E), 00029 Helsinki, Finland; 4 Center for Life Course Health Research, University of Oulu, Oulu, Finland; 5 Unit of Primary Care, Oulu University Hospital, Oulu, Finland; 6 Department of Obstetrics and Gynecology, Kuopio University Hospital, Kuopio, Finland; 7 Children’s Hospital, University of Helsinki and Helsinki University Hospital, Helsinki, Finland; 8 Research Program for Clinical and Molecular Metabolism, Faculty of Medicine, University of Helsinki, Helsinki, Finland; 9 Tampere Center for Child Health Research, Tampere University Hospital, Tampere, Finland

**Keywords:** GDM, Insulin, OGTT, Prediction, Type 1 diabetes, Type 2 diabetes

## Abstract

**Aims/hypothesis:**

The aim of this work was to examine the progression to type 1 and type 2 diabetes after gestational diabetes mellitus (GDM) in a 23 year follow-up study.

**Methods:**

We carried out a cohort study of 391 women with GDM diagnosed by an OGTT or the use of insulin treatment during pregnancy, and 391 age- and parity-matched control participants, who delivered in 1984–1994 at the Oulu University Hospital, Finland. Diagnostic cut-off levels for glucose were as follows: fasting, ≥4.8 mmol/l; 1 h, ≥10.0 mmol/l; and 2 h, ≥8.7 mmol/l. Two follow-up questionnaires were sent (in 1995–1996 and 2012–2013) to assess the progression to type 1 and type 2 diabetes. Mean follow-up time was 23.1 (range 18.7–28.8) years.

**Results:**

Type 1 diabetes developed (5.7%) during the first 7 years after GDM pregnancy and was predictable at a 2 h OGTT value of 11.9 mmol/l during pregnancy (receiver operating characteristic analysis: AUC 0.91, sensitivity 76.5%, specificity 96.0%). Type 2 diabetes increased linearly to 50.4% by the end of the follow-up period and was moderately predictable with fasting glucose (AUC 0.69, sensitivity 63.5%, specificity 68.2%) at a level of 5.1 mmol/l (identical to the fasting glucose cut-off recommended by the International Association of Diabetes and Pregnancy Study Groups [IADPSG) and WHO]).

**Conclusions/interpretation:**

All women with GDM should be intensively monitored for a decade, after which the risk for type 1 diabetes is minimal. However, the incidence of type 2 diabetes remains linear, and therefore individualised lifelong follow-up is recommended.

**Electronic supplementary material:**

The online version of this article (10.1007/s00125-020-05215-3) contains peer-reviewed but unedited supplementary material, which is available to authorised users.

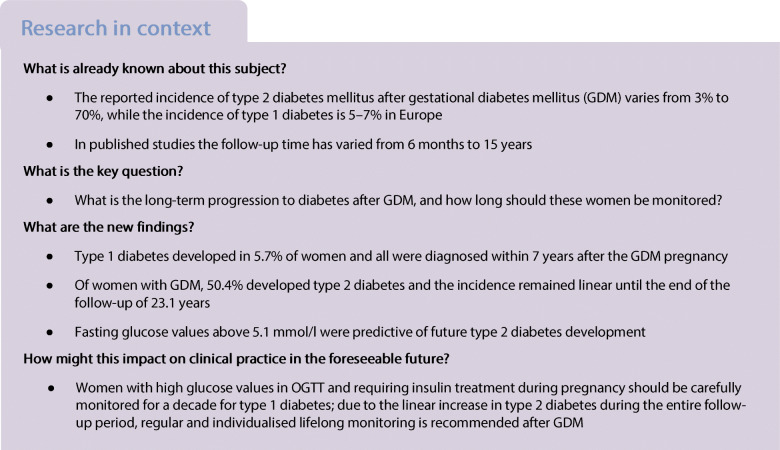



## Introduction

The prevalence of gestational diabetes mellitus (GDM) [1] and type 2 diabetes [2] is increasing worldwide and studies have shown that women with GDM are at high risk of developing diabetes later in life. However, the reported incidence of type 2 diabetes after GDM varies considerably from 3% to 70% depending on the follow-up time and ethnicity [3–5]. The overall incidence of type 1 diabetes mellitus after GDM is significantly lower, at 5–7% in Europe, but the risk increases with the number of positive autoantibodies and the duration of follow-up after pregnancy [6].

Insulin resistance increases during pregnancy because of increasing weight and adiposity combined with reduced insulin sensitivity by placental hormones. In relatively short follow-up studies (up to 15 years), high glucose levels in an OGTT, age, BMI, insulin treatment during pregnancy and a single autoantibody positivity have been predictive factors of later onset of diabetes [7–9].

We previously reported a prospective 6 year follow-up study of women with GDM and healthy control counterparts, showing that 4.6% of the GDM cohort developed type 1 diabetes and 5.3% developed type 2 diabetes, while none of the control group became diabetic [6]. We report here the results of the 23 year follow-up study of these women regarding the progression to type 1 and type 2 diabetes after GDM, including demographic, diagnostic and treatment data.

## Methods

### Study population and design

The population and design of this study has been described previously [6], and further information can be found in the electronic supplementary material (ESM) Methods. Briefly, this cohort study included 435 white women with GDM and a singleton pregnancy. The control cohort of 435 white women was pair-matched by age (±2 years), parity and date of delivery (±2 days). Both cohorts delivered at the Oulu University Hospital, Finland. Only women with GDM diagnosed by oral glucose tolerance test (OGTT) (*n* = 363) or who were treated with insulin (*n* = 28), and their matched control counterparts (*n* = 391), were included in further analysis. The cut-off values for the glucose concentrations were set according to the recommendation of the Finnish Diabetes Association: fasting, ≥4.8 mmol/l; 1 h, ≥10.0 mmol/l; and 2 h, ≥8.7 mmol/l. Any single abnormal value in the OGTT was considered diagnostic for GDM.

### Questionnaire-based follow-up

An invitation to participate in this study was sent in 1995–1996 (1–11 years after pregnancy) along with the first follow-up questionnaire and an informed consent form. The questionnaire included questions about GDM treatment (diet or insulin), pre-pregnancy weight and height, progression to diabetes, time of diagnosis and diabetes medication.

A second questionnaire was sent out in 2012–2013. Thirteen women with GDM (3.3%) and six control participants (1.5%) had died. Finally, 297 women with GDM and 297 control participants (76.0%) took part in the study. The mean follow-up period from delivery to the date of completing the questionnaire was 23.1 (range 18.7–28.8) years in the GDM cohort and 23.3 (range 18.9–30.1) years in the control cohort.

### Statistical analysis

Comparisons of baseline demographic characteristics between the groups were performed using one-way ANOVA. Kaplan–Meier survival curves were used to compare the development of type 1 or type 2 diabetes after pregnancy. To find the best predictive model for progression to type 1 and type 2 diabetes, sensitivity and specificity, receiver operating characteristic (ROC) curves were constructed with continuous glucose values. AUC was used in the classification analysis. The analyses were performed with IBM SPSS Statistics for Windows (versions 21 and 25, IBM, Armonk, NY) and RStudio (Boston, MA) software. The figures were produced using the ggplot2 (R package version 0.4.6., https://CRAN.R-project.org/package=survminer) and Adobe Illustrator (Adobe Systems, San Jose, CA).

## Results

At the end of the follow-up period, the mean ± SD age of the GDM cohort was 54.7 ± 6.4 years, and that of the control cohort was 55.3 ± 6.4 years. Body weight and BMI were higher in the GDM group than the control group, as expected (ESM Tables 1, 2). However, weight gain and increase in BMI during pregnancy was higher in the control group. The influence of age on disease progression is depicted in ESM Fig. 1 and in ESM Results.

**Fig. 1:**
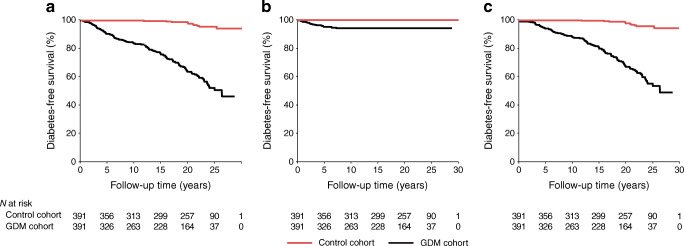
Probability of remaining free from (**a**) diabetes, (**b**) type 1 diabetes or (**c**) type 2 diabetes among women with and without GDM. Logrank *p* < 0.001 in all figure parts. Mean (95% CI) diabetes-free survival time in women with vs without GDM was as follows: diabetes, 21.5 (20.5, 22.4) years vs 29.6 (29.3, 29.9) years; type 1 diabetes, 26.7 (25.8, 27.5) years vs no occurrence of type 1 diabetes; and type 2 diabetes, 22.6 (21.7, 23.5) years vs 29.6 (29.3, 29.9) years

During the follow-up study, 53.2% of women in the GDM group developed diabetes (type 1, 5.7%; type 2, 50.4%) and 5.5% developed type 2 diabetes in the control group (Fig. 1). No-one in the control group developed type 1 diabetes. Incidence of type 2 diabetes increased linearly until the end of the study, while all the participants who developed type 1 diabetes were diagnosed during the first 7 years after pregnancy. Details of the OGTT measurements are shown in ESM Table 3.

### OGTT glucose levels in prediction of disease progression

**Fig. 2:**
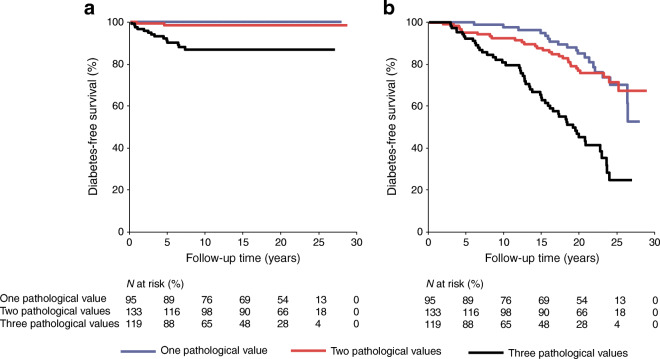
Probability of remaining free from (**a**) type 1 diabetes and (**b**) type 2 diabetes according to the number of pathological glucose values in OGTT during the GDM pregnancy. Logrank *p* < 0.001. Mean (95% CI) type 1 diabetes-free survival time was as follows: one pathological value, no occurrence of type 1 diabetes; two pathological values, 28.2 (27.5, 29.0) years; three pathological values, 21.4 (18.8, 24.0) years. Mean (95% CI) type 2 diabetes-free survival time was as follows: one pathological value, 25.0 (23.8, 26.1) years; two pathological values, 24.3 (22.9, 25.8) years; three pathological values, 17.9 (16.2, 19.6) years

Pathological glucose value at any of the three OGTT time points was associated with a shorter time to progression to type 1 or type 2 diabetes (ESM Fig. 2). Moreover, three abnormal glucose values in OGTT were most predictive of both type 1 and type 2 diabetes occurrence (Fig. 2). Altogether, 60.9% of the women with abnormal fasting glucose developed type 2 diabetes. In the ROC analyses with continuous glucose values, the most predictive single OGTT value regarding type 1 diabetes was the 2 h glucose value (AUC 0.91, sensitivity 76.5%, specificity 96.0% at a glucose level of 11.9 mmol/l). The most predictive OGTT value for type 2 diabetes was fasting glucose (AUC 0.69, sensitivity 63.5%, specificity 68.2% at a glucose level of 5.1 mmol/l) (ESM Table 4).

To dissect undiagnosed diabetes in our GDM cohort, we analysed a subgroup ‘diabetes in pregnancy’ comprising of women with fasting glucose ≥7.0 mmol/l or 2 h glucose ≥11.1 mmol/l, according to the WHO criteria [10]. Forty-eight (12.3%) women belonged to this subgroup based on their OGTT. During the follow-up period, 13 (27%) women in this subgroup developed type 1 diabetes and 22 (46%) women developed type 2 diabetes.

### Insulin treatment for GDM in prediction of disease progression

Women who received insulin treatment for GDM had a higher probability of subsequent diagnosis of both type 1 and type 2 diabetes (ESM Fig. 3). Furthermore, the time to diabetes diagnosis was longer in women without insulin treatment for GDM. Conversely, only a few women (1.2%) without insulin treatment developed type 1 diabetes. The sensitivity of insulin treatment to predict type 1 diabetes was 90.5% and specificity 64.7% (AUC 0.78) according to the ROC analyses; sensitivity as regards predicting type 2 diabetes was 56.9%, and specificity 74.6% (AUC 0.66) (ESM Table 4).

## Discussion

This 23 year prospective cohort study demonstrated that after GDM, 5.7% of women developed type 1 diabetes and approximately 50% developed type 2 diabetes. Type 1 diabetes was diagnosed less than a decade after the GDM, while the incidence for type 2 diabetes remained linear until the end of the study.

To our knowledge, this is one of the longest prospective follow-up studies on women with GDM. The majority of the diagnoses (92.8%) were made based on OGTT, albeit using capillary glucose determinations. In addition, the participation rate (76%) was remarkably high considering the long follow-up period. The main weakness of the study is the self-reported disease progression. Had OGTT been performed systematically as part of the follow-up, the incidence of type 2 diabetes might have been higher in both cohorts, as the prevalence of undiagnosed disease is reported to be as high as 20% in the general population [11]. It is very unlikely that the participants would have mixed up the type of diabetes that they have, as the diagnosis, treatment and follow-up of type 1 diabetes takes place in a specialised hospital setting while type 2 diabetes care occurs at the health-centre level. However, it is worth noting that the incidence of type 1 diabetes among young adults is higher in Finland than in other countries [12]. At the time of this study, a risk-based screening strategy for GDM was used in Finland, while nowadays nearly universal screening is recommended, and this may have underestimated the incidence of GDM.

Few prior studies compared the predictive value of single glucose levels or their combinations in OGTT regarding type 2 diabetes development. In the current study, 60.9% of the women with abnormal fasting glucose developed type 2 diabetes. Consistent with our results, three other studies reported that abnormal fasting glucose is the strongest predictor of later type 2 diabetes [13]. Conflicting results have also been reported, showing abnormal 1 h glucose or 2 h glucose to be the strongest predictor of type 2 diabetes [14]. However, the glucose load in OGTT also varied from 50 g to 75 g or 100 g among studies. In our study, each OGTT time point was independently predictive of type 2 diabetes, justifying the inclusion of the 1 h test, which is omitted in some countries. Remarkably, the identical cut-off for fasting glucose (5.1 mmol/l) that was associated with the future type 2 diabetes risk in this study, was associated with offspring risk and maternal complications in the Hyperglycemia and Adverse Pregnancy Outcomes (HAPO) study, from which the current International Association of Diabetes and Pregnancy Study Groups (IADPSG)/WHO diagnostic guidelines are derived [10, 15, 16]. Therefore, the present study further validates the use of the lower fasting glucose cut-offs for GDM diagnosis.

When examining the predictive value of OGTT glucose levels in terms of progression to type 1 diabetes, the most predictive single OGTT time point for later type 1 diabetes was the 2 h glucose concentration with a cut-off of 11.9 mmol/l, which is higher than the current diagnostic level for GDM (7.8–8.6 mmol/l). In addition, most of the women (72.9%) who fulfilled the WHO criteria for the ‘diabetes in pregnancy’ subgroup were later diagnosed with diabetes.

In the present study, insulin treatment during pregnancy was a strong predictor for both type 1 and type 2 diabetes, which is in agreement with several earlier studies [5, 6, 12, 13]. Moreover, women who had received insulin treatment for GDM developed diabetes earlier, and only a few women (1.2%) without insulin treatment developed type 1 diabetes. During the first 10 years of follow-up, the development of type 2 diabetes was rare in women without insulin treatment for GDM, but thereafter the disease progression curve was almost linear and parallel to that of women on insulin for GDM. This reflects the association of exogenous insulin treatment with the severity of an impaired glucose tolerance, also predictive of higher diabetes progression rate later in life. In addition, being over 30 years of age at the time of GDM increased the progression rate to type 2 diabetes. However, this association disappeared when the glucose levels from the OGTT were included in the same model, which suggests that abnormal glucose concentrations may be more important indicators of later type 2 diabetes.

In conclusion, women with GDM, especially those on insulin treatment, should be carefully monitored for the first decade after the pregnancy, after which the risk for type 1 diabetes becomes negligible. However, the risk for type 2 diabetes remains and warrants an individualised, lifelong follow-up.

## Electronic supplementary material

ESM (PDF 373 kb)

## Data Availability

The original data are available on request from the corresponding author. The authors declare that there are no relationships or activities that might bias, or be perceived to bias, their work.

## Abbreviations

GDM: Gestational diabetes mellitus
ROC: Receiver operating characteristic
