# Supplementary material for: Type 1 and type 2 diabetes after gestational diabetes: a 23 year cohort study
Source: Diabetologia. 2020 Jul 29;63(10):2123–8. doi: 10.1007/s00125-020-05215-3 (PMC7476985; doi:10.1007/s00125-020-05215-3)

Electronic supplementary material (ESM)

ESM Methods

Study population and design

This cohort study included all women with GDM and a singleton pregnancy but no previous GDM, type 1 diabetes or type 2 diabetes, who delivered in the Oulu University Hospital, Finland, in 1984–1994. Altogether, 435 women were enrolled. The control cohort of 435 women was pair-matched by age (±2 years), parity (nulliparous, 1–3, or more than three deliveries) and date of delivery (±2 days). All women were Caucasian. Only GDM subjects diagnosed by oral glucose tolerance test (OGTT) (n=363) or on insulin treatment (n=28), and their matched controls (n=391), were included in the analysis. In the excluded subjects (n=44), the diagnosis was based on multiple glucose measurements without a standard 75-g glucose intake, or on abnormal HbA1c values, which might refer to pre-gestational diabetes.

The indications for OGTT were glucosuria, BMI ≥25 kg/m^2, previous delivery of a macrosomic infant (≥4500 g) or expected macrosomic infant in the current pregnancy. A standard 2-h OGTT (75 g glucose load in 250 mL water) was performed after a 12-h overnight fast. Capillary whole blood samples were drawn before the OGTT at 0 min, and at 60 min and 120 min. The cut-off values for the glucose concentrations were set according to the recommendation of the Finnish Diabetes Association: fasting, ≥4.8 mmol/L; 1-hour, ≥10.0 mmol/L; and 2-hour, ≥8.7 mmol/L. Any single abnormal value in the OGTT was considered diagnostic for GDM. The blood samples were analysed using HemoCue® System (AB Leo Diagnostics, Helsingborg, Sweden) (1). The inter-assay coefficient of variation of the method was 3.8-4.0% at glucose concentration of 4.5-17.6 mmol/l.

All women diagnosed with GDM were on diet treatment (1800-2000 kcal/day). Insulin was added to the diet treatment when at least two glucose values, fasting or preprandial, were ≥5.5 mmol/l, or when 1 fasting or preprandial value was ≥5.5 mmol/l and 1 postprandial value was ≥7.8 mmol/l 1.5 hours after a meal in the 24-hour glucose profile.

All study participants signed an informed consent form. The Ethics Committee of the Northern Ostrobothnia Hospital District approved the study protocol.

ESM Results

Age

At the time of the GDM pregnancy, 40.7% of the GDM cohort were 30-year-old or younger. Morbidity in type 1 diabetes was lower and time to diagnosis was shorter in women who were 30 years or older at the time of the GDM pregnancy. In contrast, the women with a GDM pregnancy at a younger age had a lower frequency of type 2 diabetes and more of type 1 diabetes compared with older women (ESM Fig. 2). The mean age at the time of type 2 diabetes diagnosis in the younger age group was 41.9 years (SD±7.3), whereas in the older age group it was 49.9 years (SD±7.5, $P<0.005$).

ESM References

1. Ashworth L, Gibb I, Alberti KG (1992) HemoCue: evaluation of a portable photometric system for determining glucose in whole blood. Clin Chem 38:1479-82.

ESM Table 1. Demographic characteristics of the original cohorts

	Women with GDM			Control women			
	**Mean**	**N**	**SD**	**Mean**	**N**	**SD**	***P***
**Age** (years) ‡	31.3	391	5.8	31.3	391	5.7	0.862
**Weight** (kg)‡	69.5	389	14.5	61.7	391	10.4	<0.001
**Weight change** (kg) §	10.6	386	5.6	13.4	390	5.0	<0.001
**BMI** (kg/m^2)	26.3	389	5.2	22.8	391	3.5	<0.001
**BMI change** (kg/m^2) §	4.0	386	2.1	5.0	390	1.8	<0.001

‡ Age and weight at first trimester of pregnancy.

§ Weight and BMI change during pregnancy.

ESM Table 2. Demographic characteristics of the GDM and control subjects who responded to the Questionnaire 2

	Women with GDM			Control women			
	**Mean**	**N**	**SD**	**Mean**	**N**	**SD**	***P***
**Age** (years) ‡	31.1	297	5.9	31.6	297	5.5	0.369
**Weight** (kg)‡	68.3	296	13.9	61.3	297	9.8	<0.001
**Weight change** (kg) §	11.0	294	5.4	13.3	296	4.9	<0.001
**BMI** (kg/m^2)	25.8	296	5.0	22.7	297	3.3	<0.001
**BMI change** (kg/m^2) §	4.2	294	2.0	4.9	296	1.8	<0.001

‡ Age and weight at first trimester of pregnancy.

§ Weight and BMI change during pregnancy.

ESM Table 3. Characteristics of the GDM cohort.

	N=391
	% (N)
**Insulin for GDM**	
No	62.9 (242)
Yes	38.1 (149)
**GDM age**‡	
≤ 30 years	40.7 (159)
> 30 years	59.3 (232)
**Glucose levels in OGTT**§	
Abnormal fasting glucose (≥4.8 mmol/l)	67.6 (246)
Abnormal 1h glucose (≥10.0 mmol/l)	70.2 (254)
Abnormal 2h glucose (≥8.7 mmol/l)	67.3 (245)
**No. of abnormal glucose values in OGTT**ǀ	
1	26.9 (95)
2	37.7 (133)
3	33.7 (119)

§All three glucose values were measured successfully from 353 subjects.

ǀǀFasting glucose was measured successfully from 364 subjects, 1h glucose from 362 subjects and 2h glucose from 364 subjects.

ESM Table 4. Receiver operating characteristic (ROC) analyses; area under curve (AUC), sensitivity and specificity. The best predictive values in bold.

	Type 1 diabetes mellitus			Type 2 diabetes mellitus		
	AUC	Sensitivity (%)	Specificity (%)	AUC	Sensitivity (%)	Specificity (%)
**OGTT**						
0h*	0.81	100.0	45.7	**0.69**	**63.5**	**68.2**
1h†	0.76	70.6	80.3	0.58	59.6	56.4
2h‡	**0.91**	**76.5**	**96.0**	0.59	32.4	87.6
**OGTT combinations**						
0h + 1h	0.84	70.6	87.1	**0.70**	**64.1**	**70.2**
0h + 2h	**0.92**	**94.1**	**83.4**	0.69	65.4	66.5
1h + 2h	0.91	81.2	90.9	0.62	57.7	67.2
0h + 1h + 2h	0.92	93.8	83.8	0.70	74.8	59.3
Insulin for GDM	0.78	90.5	64.7	0.66	56.9	74.6
**Combination with insulin**						
0h + insulin	0.86	88.9	75.9	0.71	71.8	61.4
1h + insulin	0.85	88.2	73.2	0.67	57.3	73.8
2h + insulin	0.91	82.4	91.0	0.67	57.7	74.3
0h + 1h + insulin	0.87	82.4	80.8	0.72	75.5	60.8
0h + 2h + insulin	0.92	88.2	89.1	0.71	68.9	66.0
1h + 2h + insulin	0.91	87.5	86.8	0.69	80.6	52.3
0h + 1h + 2h + insulin	0.92	87.5	89.9	0.72	68.6	67.7
**Insulin for GDM**	0.78	**90.5**	64.7	0.66	56.9	74.6

*Optimal level of fasting glucose for predicting type 1 diabetes mellitus was 4.9 mmol/l and for type 2 diabetes mellitus 5.1 mmol/l.

†Optimal level of 1h glucose for predicting type 1 diabetes mellitus was 12.0 mmol/l and for type 2 diabetes mellitus 10.9 mmol/l.

‡Optimal level of 2h glucose for predicting type 1 diabetes mellitus was 11.9 mmol/l and for type 2 diabetes 10.3 mmol/l.

ESM Figure 1. Probability of remaining nondiabetic according to age during the GDM pregnancy. A. type 1 diabetes, p=0.015; B. type 2 diabetes, p<0.001.

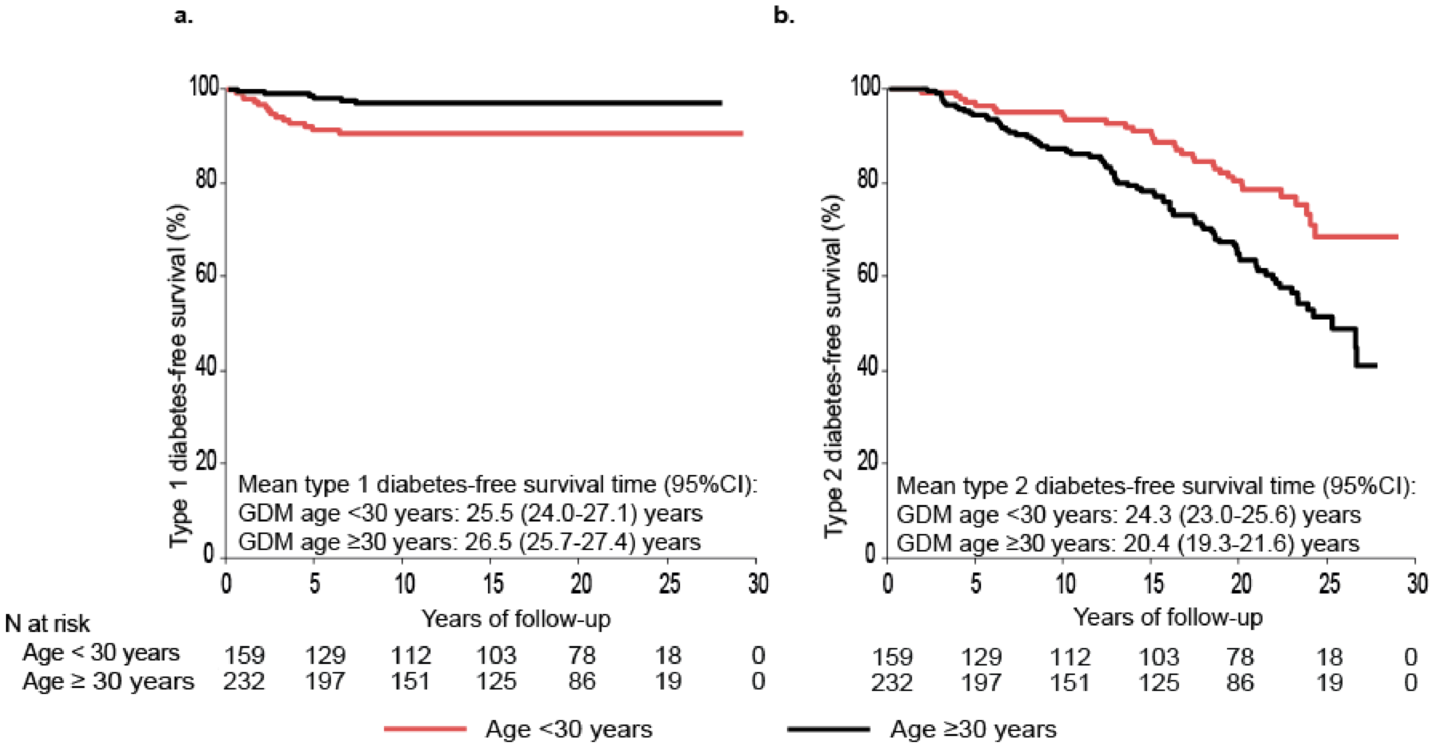

ESM Figure 2. Probability of remaining non-diabetic according to pathological glucose values in OGTTs (each value separately) during GDM pregnancy. A. type 1 diabetes (0-h p=0.001; 1-h p=0.089; 2-h p=0.003); B. type 2 diabetes (0-h p<0.001; p=0.110; p=0.028).

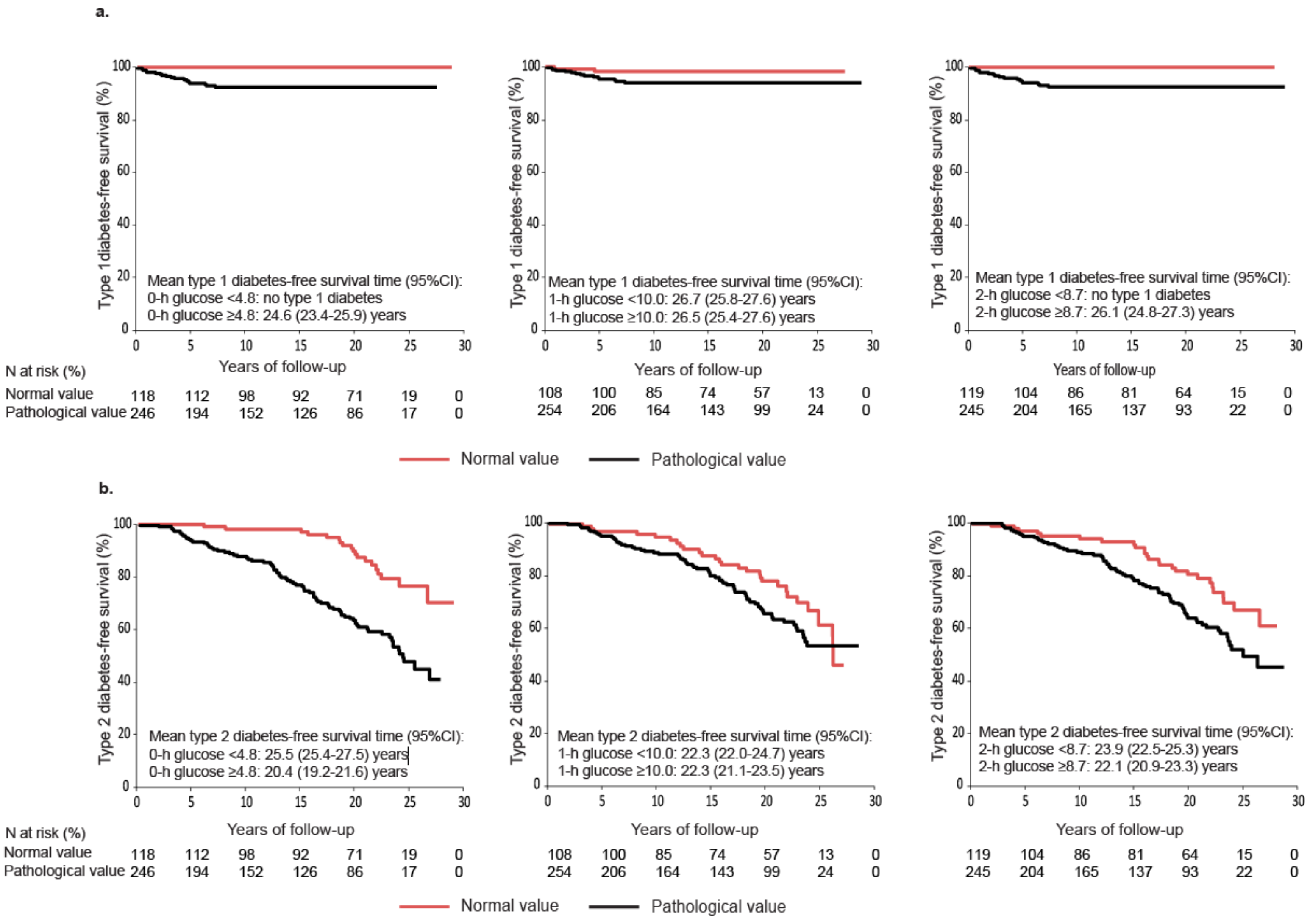

ESM Figure 3. Probability of remaining non-diabetic according to treatment for GDM (insulin vs. non-insulin). A. type 1 diabetes, p<0.001; B. type 2 diabetes, p<0.001.

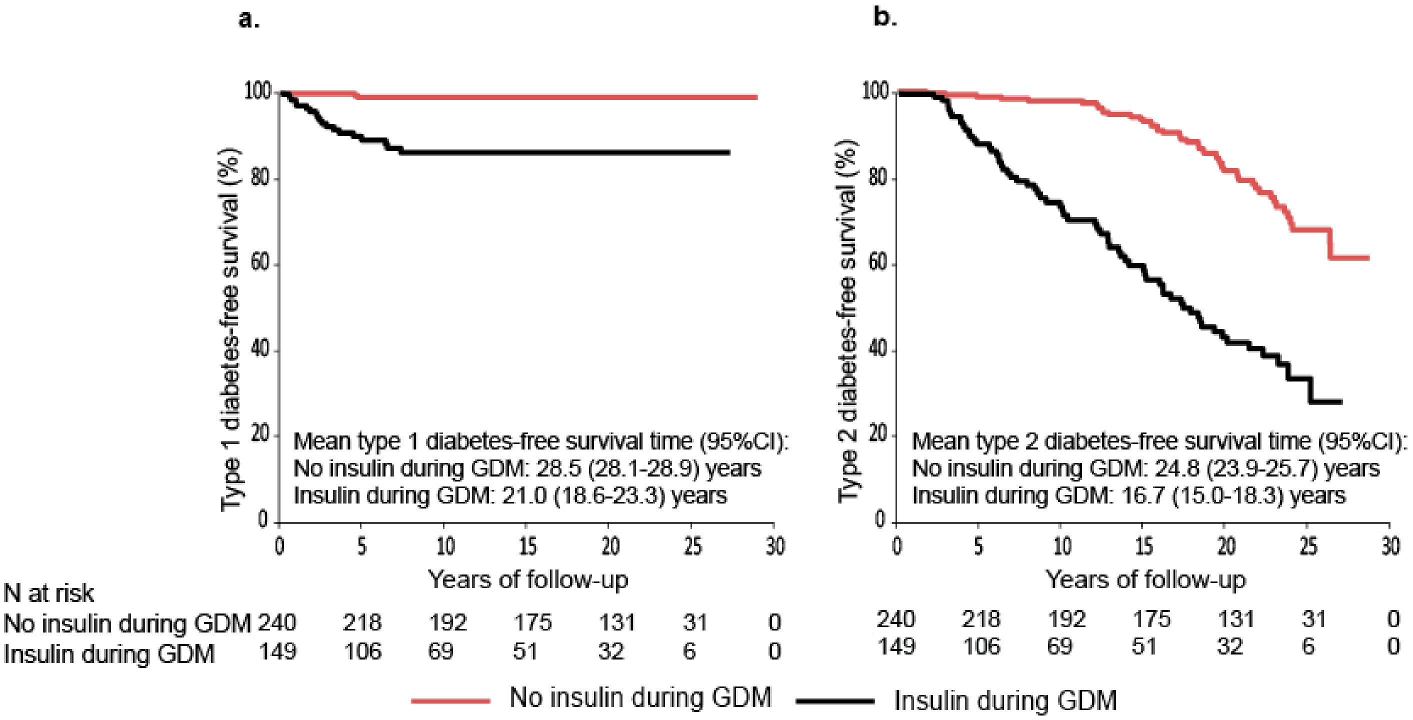

Supplement: Supplementary file 1 — (PDF 373 kb) [file 125_2020_5215_MOESM1_ESM.pdf]
